# Supplementary figures and images for: A predicted structural interactome reveals binding interference from intrinsically disordered regions
Source: PLoS Comput Biol. 2026 Jan 22;22(1):e1013899. doi: 10.1371/journal.pcbi.1013899 (PMC12854418; doi:10.1371/journal.pcbi.1013899)

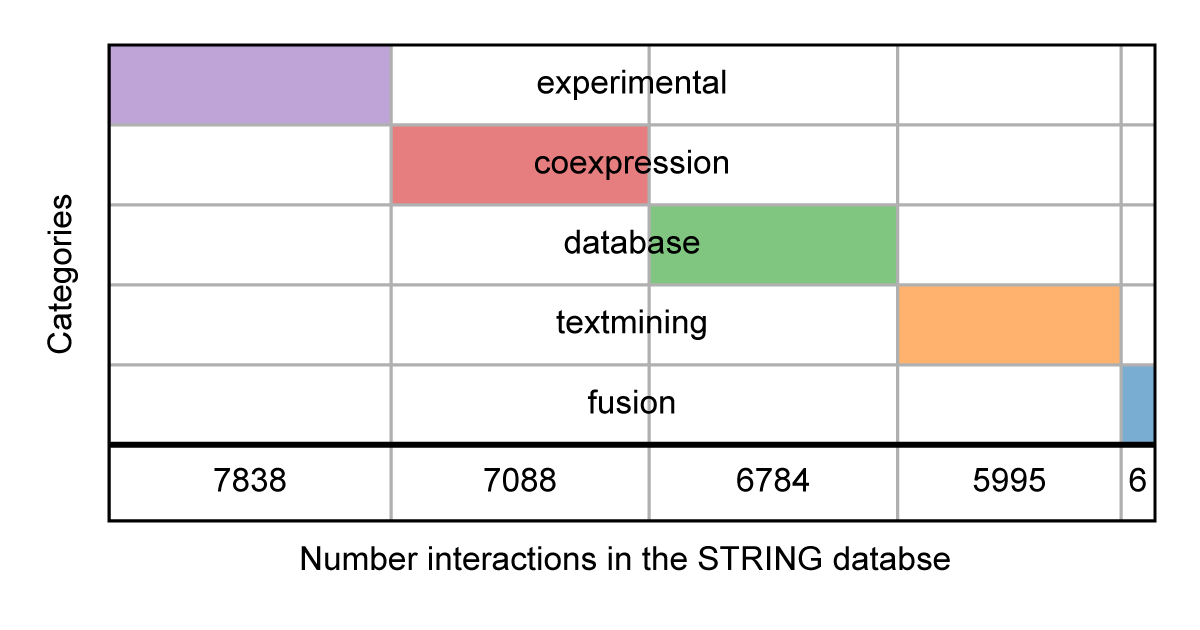

Supplement: S1 Fig — (TIF) [file pcbi.1013899.s001.tif]

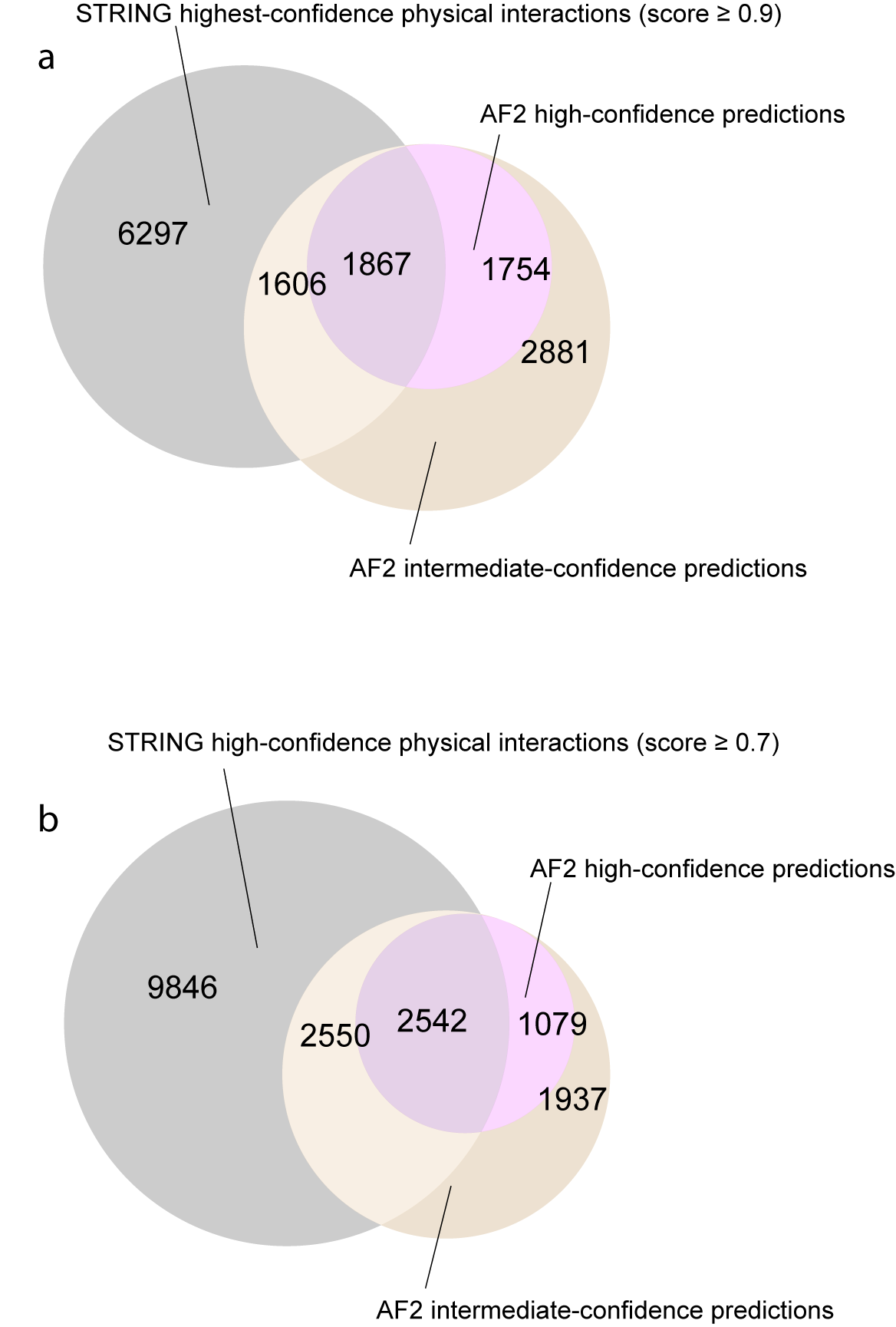

Supplement: S2 Fig — The overlap between AlphaFold predictions and the highest-confidence STRING physical interactions (STRING physical confidence score ≥ 0.9). b). The overlap between AlphaFold predictions and the high-confidence STRING physical interactions (STRING physical confidence score ≥ 0.9). (TIF) [file pcbi.1013899.s002.tif]

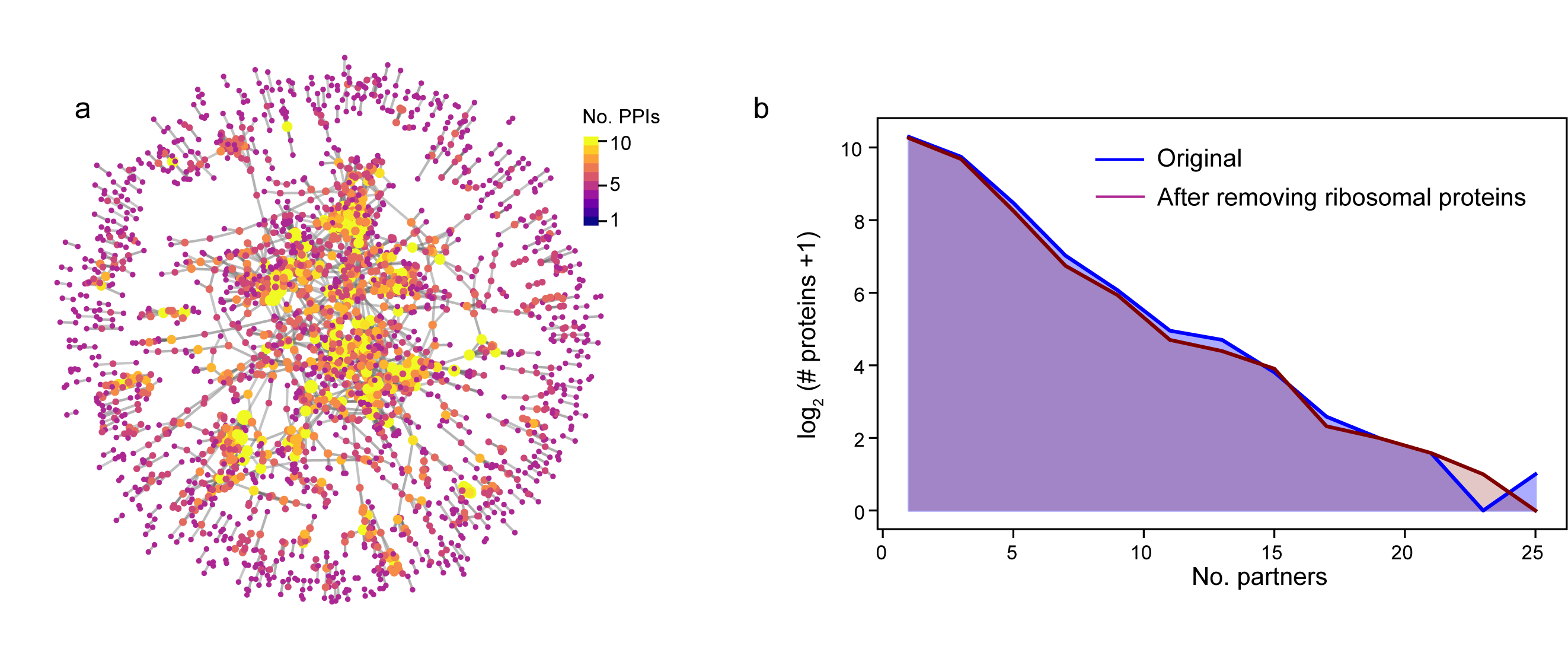

Supplement: S3 Fig — (a) The modified network, obtained after removing 154 ribosomal proteins, including both cytoplasmic and mitochondrial, retains a similar overall layout to the original network (Figure 1b in the main text). (b) The modified network also shows a comparable connectivity distribution to that of the original. The connectivity distribution represents the number of proteins that have a given number of interaction partners. Note that the Y axis is in log scale, log2(Number of proteins + 1). (TIF) [file pcbi.1013899.s003.tif]

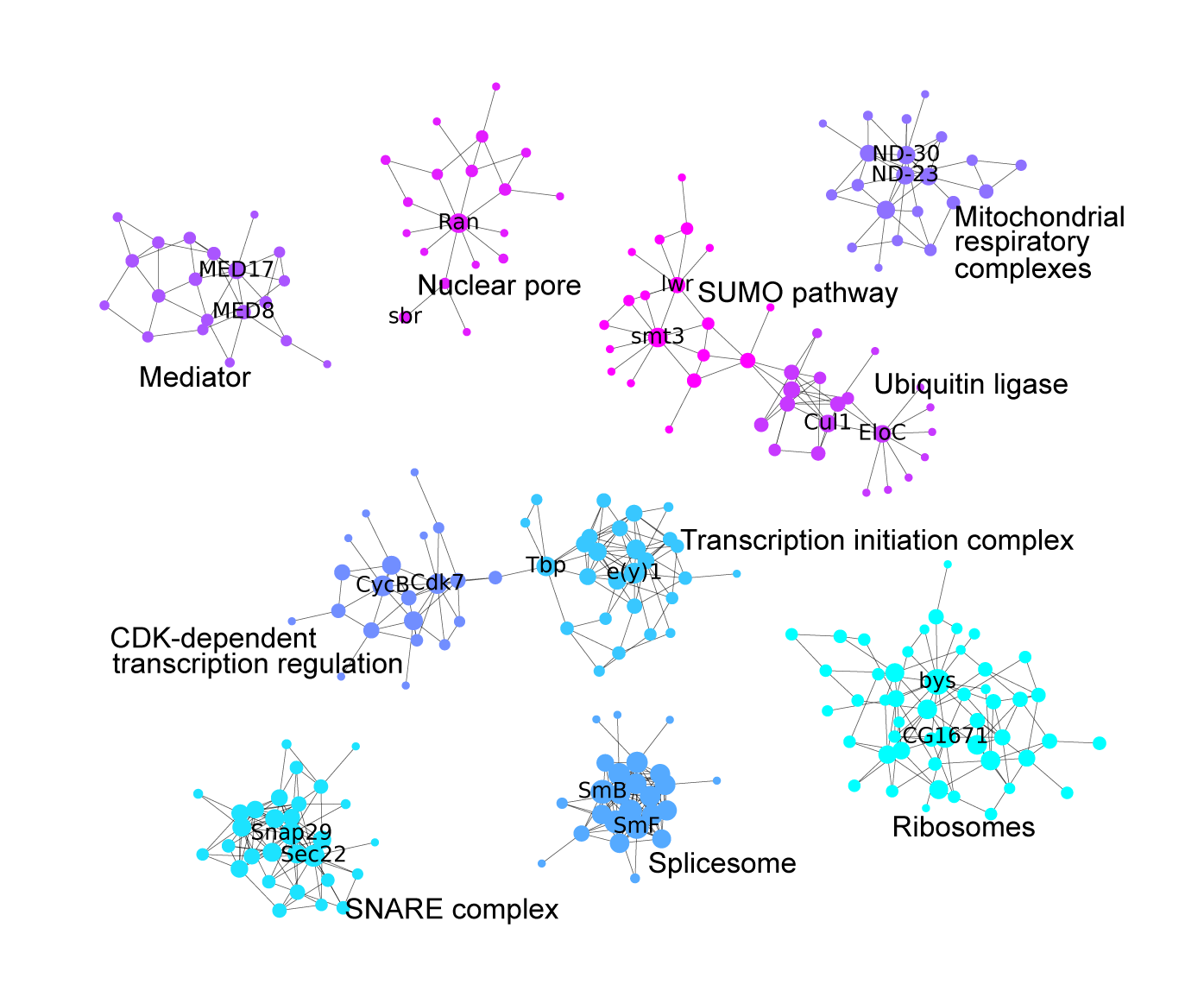

Supplement: S4 Fig — Each cluster corresponds to an essential protein complex or a crucial cellular pathway. (TIF) [file pcbi.1013899.s004.tif]

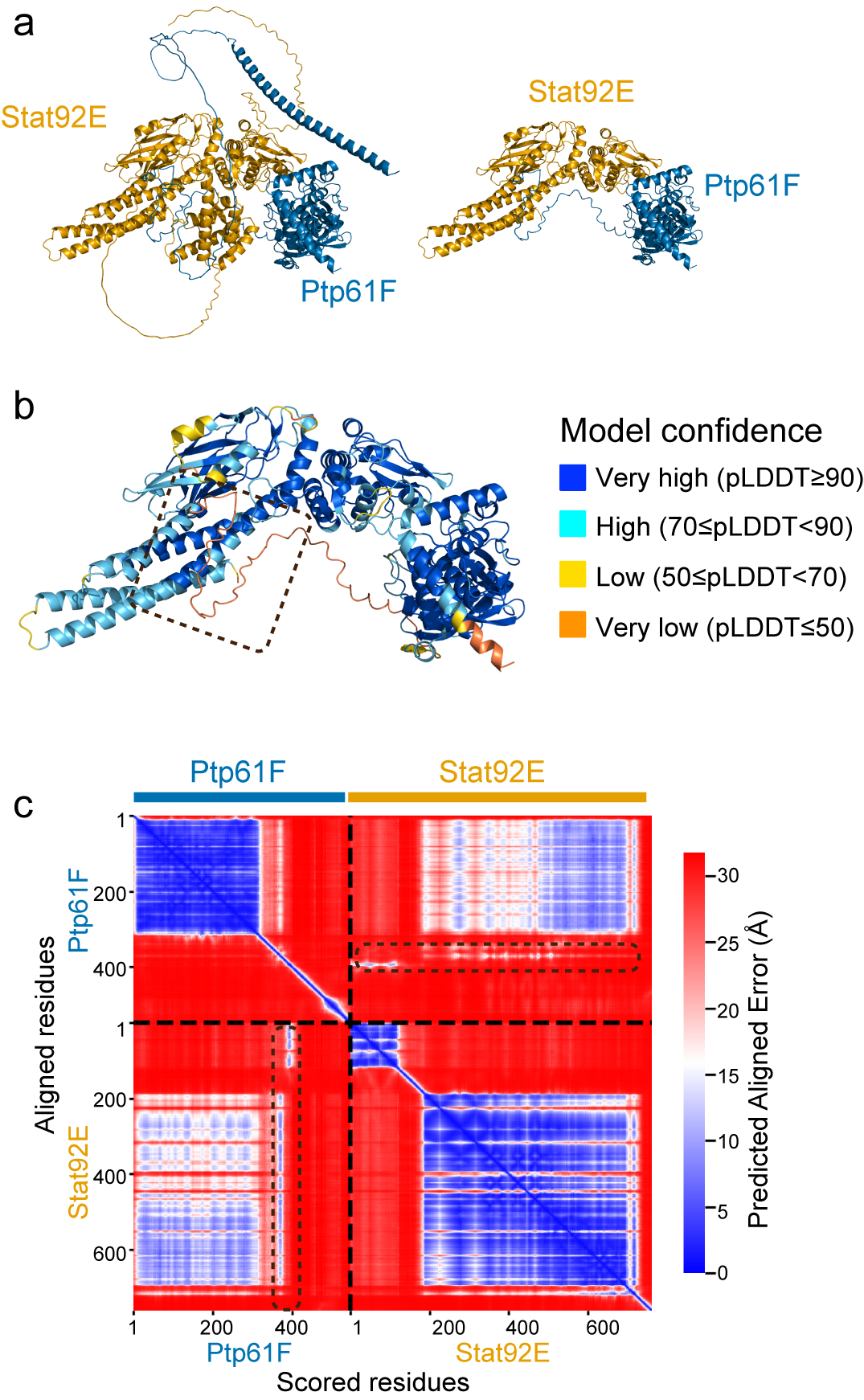

Supplement: S5 Fig — (a) Left: full-length predicted model. Right: truncated model with the C-terminus of Ptp61F (residues 380–548), the N-terminus of Stat92E (residues 1–187), and the C-terminus of Stat92E (residues 696–961) removed for clearer visualization. (b) Model confidence (pLDDT) for the truncated model shown in (a). The dashed-line box highlights a region where binding involves a low-confidence segment of Ptp61F (residues 360–370). (c) PAE matrix. The blue off-diagonal region highlighted by the dashed-line box corresponds to the potential binding region indicated in (b). (TIF) [file pcbi.1013899.s005.tif]

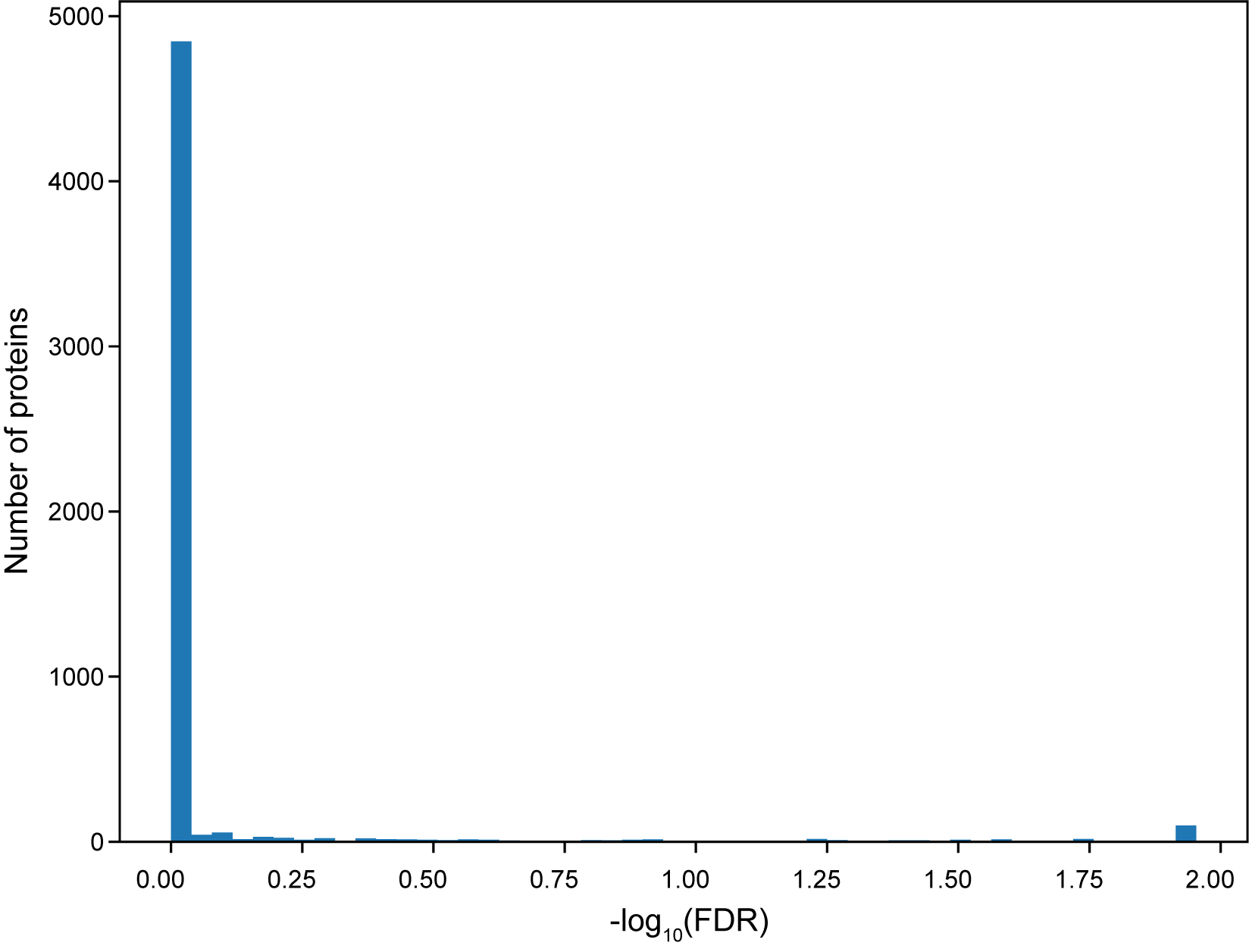

Supplement: S6 Fig — To determine whether prediction failures were randomly distributed across proteins, we performed a permutation test in which success labels were randomly reassigned 5,000 times while maintaining the total number of successful predictions. For each protein, empirical p-values were computed from the permutation distribution of success fractions and corrected for multiple testing using the Benjamini-Hochberg false discovery rate (FDR) method. The resulting FDR distribution shows that most proteins have FDR values close to 1, with only a small number of outliers, indicating that prediction failures were largely random rather than systematically associated with particular proteins or interaction types. (TIF) [file pcbi.1013899.s006.tif]
