# Supplementary material for: A predicted structural interactome reveals binding interference from intrinsically disordered regions
Source: PLoS Comput Biol. 2026 Jan 22;22(1):e1013899. doi: 10.1371/journal.pcbi.1013899 (PMC12854418; doi:10.1371/journal.pcbi.1013899)
Supplement: Attachment — Submitted filename: Review PLOS notes.pdf [file pcbi.1013899.s016.pdf]

To the authors

This work uses Alphafold 2 to predict protein pairs from the STRING database for Drosophila melanogaster to gain more insights into the structural details of their interaction. Based on pre-filtering, they predict 27,711 pairs and publish their results in an interactive web server (https://jhpanda.github.io/DmelPPI_web/), which can be filtered by protein names and/or specific pairs. They then compared the alphafold predictions with the STRING confidence scores for physical interaction to identify highly confident protein interactions and regions that undergo structural rearrangements, intrinsically disordered regions (IDR) involved in binding, and conditionally folded interfaces between the proteins. They analyzed their dataset, showing connections between proteins, giving insights into the proportion of differently defined IDRs, putting some in an evolutionary context, and discussing examples for a possible binding mechanism (differences in secondary structure between monomer prediction and complex prediction). The results are (mostly) clearly prepared and in a logical order.

During the review, only a few examples (e.g., “Ku80 Irbp”) from the web server were accessible, while other links were inactive, and structures were not visible. Crucially, the inability to download models or associated data prevents independent analysis and severely limits the resource's usability. The dataset appears to overlap significantly with the existing FlyPredictome database. Nevertheless, I appreciate the IDR plots and analysis.

The associated GitHub repository lacks the necessary documentation, comments, or instructions for other researchers to use the scripts or reproduce the results. I would greatly appreciate it if the repository were fully documented, including details on inputs, scripts, and the entire workflow.

General things

- The paper “Enhanced Protein-Protein Interaction Discovery via AlphaFold-Multimer” (https://doi.org/10.1101/2024.02.19.580970) also investigated the Drosophila protein-protein interaction and created a database called “FlyPredictome.” I assume there is a huge overlap between these two sources. What is the benefit of using the created database apart from the already existing one? For the FlyPredictome database, there are many more examples, e.g., Ku80 (44 vs. 1).
- What is the actual deviation between the corresponding PDB structures for the prediction where they exist? It is hard to evaluate the correctness of the predictions just by showing an overlay of all, especially when these are large complexes.

- For the prediction of all protein-protein pairs, only 13.1% of them show high confidence in pDockQ (> 0.5). In the result section, the author concludes that only “a relatively small percentage of the previously reported functional associations may have direct physical interactions”. Based on a comparison with solved structures (e.g., https://doi.org/10.1038/s41594-022-00910-8) others say that with these cutoffs, around 80% of models can be assumed to be correct. What does this mean for your own models?

Predictions / AlphaFold

- How many structures are used from the structural database? Please add IDs for all models in the supplementary materials for reproducibility.
- How many models were generated for each protein-protein pair? Were random seeds used?
- You tried to predict 34,940 protein pairs and obtained 27,711. Where are the other models? Was there a pLDDT cutoff chosen for the overall prediction? If yes, which one?
- What do you mean by “full version of AlphaFold2 multimer”? Why not use Alphafold3, Boltz, or Chai?
- Another study (https://doi.org/10.1101/2024.02.19.580970), which examined Drosophila protein predictions, introduced LIS to enhance sensitivity in detecting PPIs for flexible and small interfaces based on pAE values. And because you examined later short segments for conditional folds, I think this is reasonable because pDockQ is only based on the pLDDT values of the interface residues.
- For AlphaFold-disorder, disordered regions are also smoothed using a sliding window approach for the RSA calculation. Do you use these values in the calculation of the one without smoothing? GitHub mentioned the smoothing window, but which window size was used to calculate the monomer/complex predictions?

Thoughts about the webpage

- What exactly does “acceptable” mean in the PPI column? When I search, e.g., rab5, a result (with CG32350) pops up showing an ipTM of 42.3 and a pDockQ of 0.338. A score of ipTM below 0.6 suggests a failed prediction. Is this just based on the pDockQ score? Unfortunately, I could not look at the models, because the web server was not showing the results.

- For the ID 7227.FBpp0080322_7227.FBpp0081861, the pAE matrix is very off-diagonal, and it does not display the correct values. Because I am limited in the number of IDs that I can test, I'm not aware how often that occurs (tested on 22.09)

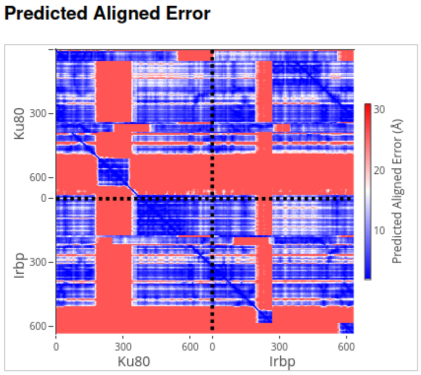

Reproducibility and Code

- Provide comprehensive instructions on how to use the scripts, detailing the required input data, the function of each script, and the overall workflow
- The repository should include all necessary files (or at least Zenodo links), such as the input sequences, the predicted models, and the scripts used to process the data and generate the figures clearly and logically. I think GitHub would benefit a lot from much clearer instructions

Additional things I've seen

- Line 74/85: In both ISD cutoffs are mentioned for highly disordered regions, but in one line, you write “disordered if [...] ISD < 0.5” and in the other “ high structural disorder ISD > 0.5”
- Line 84, 247, 248 (maybe more): PLDDT -> pLDDT
- Fig. 1a: Please label the x-axis (confidence score?)
- Fig. 1b: I know it is a network of possible protein-protein interactions, but for me as a reader, it does not show anything informative besides the network size and a lot of points over each other.
- Fig. 2a/b: Three median-sized connected subnetworks are mentioned in the text (l. 202-203), but I see only two in the figure. In addition, even though the names in the networks are interesting, there is too much information in them, and in some cases, I'm not sure which label belongs to which.
- Fig 2c/d: Choosing red and blue for the coloring could confuse the reader because the matrix in e uses the same colors for another purpose.
- Fig 2c/d: Using red and green is unfortunate for colorblind readers.
- Fig 2c/d: It would be nice to have pLDDt colored structures or only show one structure, because it is hard/impossible for the reader to identify high confidence predictions in this complex
- Fig 2c/d: Why are there residues indicated in the structures of Ran/Nup50. They are not mentioned in the text. Should they interact with each other?

- Fig 2d/e: Please change the numbering of the pAE matrix to match the number of the residues mentioned in the structure/text to make it clear which parts are meant. Or color some parts differently to highlight the interface.
- Fig 3c: Which one is Ku80 and which Irbp?
- Fig 3d: In the left panel, the Ku80 residues from 246-272 are highlighted, but in the text, 245-284 are mentioned. I
