# Supplementary material for: A predicted structural interactome reveals binding interference from intrinsically disordered regions
Source: PLoS Comput Biol. 2026 Jan 22;22(1):e1013899. doi: 10.1371/journal.pcbi.1013899 (PMC12854418; doi:10.1371/journal.pcbi.1013899)
Supplement: Attachment — Submitted filename: Peng_response.docx [file pcbi.1013899.s017.docx]

**Respond to reviewers’ comments**

**Reviewer #1**

This work uses Alphafold 2 to predict protein pairs from the STRING database for Drosophila melanogaster to gain more insights into the structural details of their interaction. Based on pre-filtering, they predict 27,711 pairs and publish their results in an interactive web server (https://jhpanda.github.io/DmelPPI_web/), which can be filtered by protein names and/or specific pairs. They then compared the alphafold predictions with the STRING confidence scores for physical interaction to identify highly confident protein interactions and regions that undergo structural rearrangements, intrinsically disordered regions (IDR) involved in binding, and conditionally folded interfaces between the proteins. They analyzed their dataset, showing connections between proteins, giving insights into the proportion of differently defined IDRs, putting some in an evolutionary context, and discussing examples for a possible binding mechanism (differences in secondary structure between monomer prediction and complex prediction). The results are (mostly) clearly prepared and in a logical order. During the review, only a few examples (e.g., “Ku80 Irbp”) from the web server were accessible, while other links were inactive, and structures were not visible. Crucially, the inability to download models or associated data prevents independent analysis and severely limits the resource's usability. The dataset appears to overlap significantly with the existing FlyPredictome database. Nevertheless, I appreciate the IDR plots and analysis. The associated GitHub repository lacks the necessary documentation, comments, or instructions for other researchers to use the scripts or reproduce the results. I would greatly appreciate it if the repository were fully documented, including details on inputs, scripts, and the entire workflow.

Response: We thank the reviewer for the insightful comments and constructive suggestions.

Our datasets are different from FlyPredictome, as our study has a different emphasis (explained below), despite using similar tools. Both datasets will benefit researchers in broader computational and experimental fields, especially those who study insect biology. We have also updated the GitHub documentation (https://github.com/LiZhaoLab/DmelPPI).

General things

- The paper “Enhanced Protein-Protein Interaction Discovery via AlphaFold-Multimer” (https://doi.org/10.1101/2024.02.19.580970) also investigated the Drosophila protein-protein interaction and created a database called “FlyPredictome.” I assume there is a huge overlap between these two sources. What is the benefit of using the created database apart from the already existing one? For the FlyPredictome database, there are many more examples, e.g., Ku80 (44 vs. 1).

Response: We noted and cited the other bioRxiv paper on predicting protein-protein interactions in Drosophila. There are several aspects in which our datasets differ from FlyPredictome and combined, would be of benefit to scientists. (1) Our datasets are different from FlyPredictome. FlyPredictome used high-confidence physical interactions from IP-MS, Y2H, and curated lists of physical interactions. In our manuscript, we used PPIs from the STRING database, which includes both physical interactions, detected experimentally or inferred from homology, and functional associations, derived from co-expression, genomic context, etc. We include functional associations because previous literature found that proteins with functional associates have a higher chance of interacting physically than by chance. We wanted to know, on the proteome scale, how these functional associations can contribute to biologically meaningful interactions. (2) We asked different scientific questions. FlyPredictome highlighted the new “LIS” metric for evaluating AlphaFold multimer predictions. In our manuscript, we systematically examined the interactions from the STRING database, including those categorized as functional associations. We emphasized how functional associations contribute to the highly confident predictions. Another emphasis of our manuscript is the analysis of interaction details. We specifically examined how disordered regions contribute to protein-protein interactions on a proteome-wide scale. We also showed examples of how the IDR interfaces evolve, which is not the focus of the FlyPredictome.

Our dataset could benefit readers in several ways. (1) From our analysis, users could search for not only direct interactions supported by high-confidence experimental supports but also direct interactions that are previously categorized as functional associations. In this way, the dataset could serve as a useful resource and hypothesis generator for future studies. (2) Our analysis provides a detailed structural analysis of the interactions, especially those involved in intrinsic disorders. These data could help to dissect detailed binding mechanisms of specific intrinsic disordered proteins or proteins with disordered regions when interacting with their protein partners. (3) Our dataset could facilitate comparative analyses across species to investigate how IDR interfaces evolve and contribute to novel protein-protein interactions.

For the specific protein Ku80 (FBpp0080322), the STRING database (version 11.5) lists only three partners with the highest STRING confidence > 0.9, including Irbp (FBpp0081861), DNALig4 (FBpp0073678), and CG12728 (FBpp0070824). Among the three partners, DNALig4 has a sequence length of 918, combined with Ku80’s length of 699, the complex is over 1600 amino acids, which is beyond the capability of the GPU nodes used in our study. For CG12728, it is not a representative protein, and the representative protein in this cluster is CG32756 (FBpp0070825), which does not interact with Ku80 in the STRING database (version 11.5).

We wanted to emphasize that our analysis is to understand how functional associations contribute to the prediction of protein-protein interactions and how disordered bindings contribute to protein-protein interactions at genomic scales. The smaller number of interactions for Ku80 does not affect our conclusion. However, we recognize that this represents a limitation of our study, and we add additional discussions on this point. We included addition discussion in the revised manuscript below for your reference.

“Note that in this study, we used cluster-based representative proteins from the STRING database. This can lead to discrepancies when a non-representative isoform is annotated as the interacting partner. Additionally, interacting partners form complexes that exceed the sequence-length limits of the computational resources available, preventing us from predicting the interactions between those pairs. These constraints may result in a reduced number of predicted interactions for a small subset of specific proteins. While these instances do not affect the overall conclusions of our study, they highlight an important limitation inherent to both the available functional annotations and current computational capabilities. Future work incorporating larger computational resources and improved functional annotations may help address these gaps”

- What is the actual deviation between the corresponding PDB structures for the prediction where they exist? It is hard to evaluate the correctness of the predictions just by showing an overlay of all, especially when these are large complexes.

Response: Thank you for this question. We have now included TM-score, a widely used metric for assessing structural similarity. Specifically, we used FoldSeek-Multimer to identify similar protein complexes in the PDB, and then used US-align to re-evaluate the TM-scores. We have also added the US-align procedures to the Methods section.

- For the prediction of all protein-protein pairs, only 13.1% of them show high confidence in pDockQ (> 0.5). In the result section, the author concludes that only “a relatively small percentage of the previously reported functional associations may have direct physical interactions”. Based on a comparison with solved structures (e.g., https://doi.org/10.1038/s41594-022-00910-8) others say that with these cutoffs, around 80% of models can be assumed to be correct. What does this mean for your own models?

Response: Based on the comparison with solved structures, the high-confidence interactions obtained in our study are likely to be meaningful in cells. We indeed observed that most of the high-confidence interactions have median or higher STRING physical confidence. We also observed that a non-negligible portion of the high-confidence interactions have low STRING physical confidence and are merely functional associations. This finding supports the idea that, for future large studies, it is important not to overlook functionally associated protein pairs.

As for the percentage of high-confidence interactions across all protein-protein pairs, we believe several factors may contribute, as discussed in our manuscript. (1) Some other interactions have pDockQ greater than 0.23, of which around 70% were proposed to be well modeled. There are many with ipTM > 50 and pAE < 10.0 Å. It is important to note that these interactions do not show high confidence, but may still be meaningful. Since the accuracy of these interactions may decrease, we chose not to include them in a detailed structural analysis in the main text. However, we did not discard these interactions; we included them on our website. On the website, users can explore their interaction details.

Predictions / AlphaFold

- How many structures are used from the structural database? Please add IDs for all models in the supplementary materials for reproducibility.

Response: For the structural database, there are 192586 mmcif files. We used AlphaFold Multimer version 2.30, and the structural database provided by this version was PDB structures with a release date before 2021-09-30. We have added the IDs used in S5 Appendix and our GitHub repo (<https://github.com/LiZhaoLab/DmelPPI>).

- How many models were generated for each protein-protein pair? Were random seeds used?

Response: For each prediction, we generated five models with random seeds. We retained the first-ranked model for further structural analysis. We have clarified this in the Methods section as follows: “For each prediction, we generated five models using different random seeds, and the best-scoring prediction was selected as the final model.”

- You tried to predict 34,940 protein pairs and obtained 27,711. Where are the other models? Was there a pLDDT cutoff chosen for the overall prediction? If yes, which one?

Response: While we aimed to generate structure predictions for all 34,940 protein–protein pairs, a subset of runs (7,229 pairs) encountered memory-related failures during the AlphaFold2 prediction process. The memory-related failures were likely caused by the total sequence length, as we used a mixed A10 and L40 GPU nodes. To support this, we performed a logistic regression to determine whether larger protein pairs (with longer total sequence length) are more likely to be missing in AF2 multimer predictions. Our logistic regression analysis revealed a highly significant negative relationship between total sequence length and prediction success (β = −0.0011, z = −30.4, p = 8.946e-207), indicating that longer protein pairs were substantially more likely to fail due to memory limitations during AlphaFold2 prediction.

Further, to ensure that these missing pairs did not introduce systematic bias into our analysis, we performed a permutation test to evaluate whether prediction failures were randomly distributed across proteins:

1. Let N = 34,940 be the total number of attempted protein pairs.
2. Label the n = 27,711 successfully predicted pairs as “success = 1” and the remaining N ­– n = 7,229 as “success = 0.”
3. Compute the observed success fraction for each protein (number of successful predictions / total pairs).
4. Randomly permute the success labels 5,000 times while keeping the total number of successes constant.
5. For each protein, record the success fractions in the permuted samples and compute an empirical p-value as the proportion of permutations with a success fraction greater than or equal to the observed value.
6. Adjust the resulting empirical p-values for multiple testing using the Benjamini–Hochberg false discovery rate (FDR) method.

The permutation results showed that most proteins had FDR values close to 1 (which means -log10(FDR) near 0), indicating that the missing predictions were largely random and not systematically biased toward specific proteins or interaction types. Therefore, these missing pairs are unlikely to affect our conclusions. We have included this analysis and the FDR distribution plot in the Supplementary Information to clarify this point. We have also attached the distribution plot below for your reference.

**S6 Fig. Permutation analysis indicates random distribution of prediction failures**. In our analysis, a total of 34,940 predictions were attempted, of which 27,711 were successfully predicted and 7,229 failed due to memory limitations. To determine whether prediction failures were randomly distributed across proteins, we performed a permutation test in which success labels were randomly reassigned 5,000 times while maintaining the total number of successful predictions. For each protein, empirical p-values were computed from the permutation distribution of success fractions and corrected for multiple testing using the Benjamini-Hochberg false discovery rate (FDR) method. The resulting FDR distribution shows that most proteins have FDR values close to 1 and thus -log10(FDR)s close to 0, with only a small number of outliers, suggesting that prediction failures were largely random rather than systematically associated with particular proteins or interaction types.

- What do you mean by “full version of AlphaFold2 multimer”? Why not use Alphafold3, Boltz, or Chai?

Response: By “full version of AlphaFold2 multimer,” we refer to the use of the complete sequence database (as described in the original AlphaFold2 publication) for multiple sequence alignment (MSA) generation, rather than a reduced or curated subset.

At the time this project was initiated (the project took >18 months to run and more time to analyze and write), AlphaFold3, Boltz, and Chai had not yet been released. Given our limited computational resources, we focused on AlphaFold2 multimer, which has been widely validated and shown to achieve comparable accuracy to these more recent models for many protein-protein complex predictions.

- Another study (https://doi.org/10.1101/2024.02.19.580970), which examined Drosophila protein predictions, introduced LIS to enhance sensitivity in detecting PPIs for flexible and small interfaces based on pAE values. And because you examined later short segments for conditional folds, I think this is reasonable because pDockQ is only based on the pLDDT values of the interface residues.

Response: Thank you to the reviewer for bringing up the new LIS metric. In our original manuscript, we cited the study, and we believed that “By designing sophisticated, dedicated metrics, we may be able to distinguish true interactions at high precision (18, 24, 47, 55, 56, 61, 62).” We agree with the reviewer that incorporating LIS could help detect more IDR interfaces. Thus, to provide users with more qualitative insight into the predictions, we have computed the iLIS metric, as recommended by the authors (https://github.com/flyark/AFM-LIS), for each prediction and have updated it in Supplementary data 1. Accordingly, we have cited the most recent publication on the newer iLIS metric in the revised manuscript. We found that the iLIS metric shows a strong positive correlation with the pDockQ metric (Figure R1), suggesting that both capture high-confidence protein–protein interactions. Moreover, for all predicted interactions with iLIS ≥ 0.223, the recommended threshold for high-confidence interactions, the minimal PAE values were consistently below 10 Å. All such interactions were included in our analysis and are available through our interactive web resource.

We also would like to emphasize that in this manuscript, by using pDockQ, we showed the importance of IDR in protein-protein interactions and the evolutionary patterns of the IDR interfaces. In addition, in an early benchmark (Burke et al, 2023), pDockQ>0.5 shows an accuracy of 80%. From this perspective, the pDockQ metric is already enough to support our analysis and conclusions.

Figure R1. Correlation between iLIS (left column) and pDockQ (right column) with other metrics, including ipTM, and minimal PAE values.

Figure R2. Scatter plot of minimal PAE values and ipTM for predicted interactions with iLIS ≥ 0.223. From the scatter plot, we can see that with iLIS ≥ 0.223, the mPAE values were consistently under 10 Å.

- For AlphaFold-disorder, disordered regions are also smoothed using a sliding window approach for the RSA calculation. Do you use these values in the calculation of the one without smoothing? GitHub mentioned the smoothing window, but which window size was used to calculate the monomer/complex predictions?

Response: Yes, we used a sliding window approach. We used a sliding window size of 25, and a cutoff of RSA of 0.581. We have now added the detailed parameters in Methods.

Thoughts about the webpage

- What exactly does “acceptable” mean in the PPI column? When I search, e.g., rab5, a result (with CG32350) pops up showing an ipTM of 42.3 and a pDockQ of 0.338. A score of ipTM below 0.6 suggests a failed prediction. Is this just based on the pDockQ score? Unfortunately, I could not look at the models, because the web server was not showing the results.

Response: We thank the reviewer for this thoughtful observation. The term “acceptable” means intermediate-confidence interactions according to pDockQ (≥0.23). We have now added the notes for different terms in the PPI column on the website below the master table for clarification.

The rab5-CG32350 model indeed shows an ipTM of 0.423 and a pDockQ of 0.338, suggesting intermediate confidence in the predicted interface. The threshold of ipTM > 0.6 is based on AlphaFold-Multimer benchmark analyses (Evans et al., 2021), where ipTM > 0.6 generally corresponds to DockQ values considered “acceptable” under CAPRI criteria.

However, we wanted to mention that ipTM, while useful, is a heuristic derived from inter-chain alignment confidence and is known to correlate less strongly with experimental DockQ scores than pDockQ (Bryant et al., 2022, Bioinformatics; Burke et al., Nature Structural & Molecular Biology, 2023). The pDockQ score of 0.338, therefore, still supports a relatively weak but potentially biologically relevant interface.

Moreover, Rab5 and CG32350 have known or inferred functional connections in endocytic trafficking, and our goal was to provide a comprehensive resource rather than to restrict analyses solely to high-confidence complexes. Thus, we open sourced the model of Rab5-CG32350 and marked it as “acceptable” rather than “high confident” in the master table. Similarly, we included interactions in the table if pDockQ < 0.23, but if iptm ≥ 50 or minimal inter-chain PAE ≤ 10.0 Å and marked them as “possible”.

We have now provided access to all models at an updated site: <https://melppi.github.io>.

- For the ID 7227.FBpp0080322_7227.FBpp0081861, the pAE matrix is very off-diagonal, and it does not display the correct values. Because I am limited in the number of IDs that I can test, I'm not aware how often that occurs (tested on 22.09)

Response: We apologize the inconvenience. Now we have updated our site (<https://melppi.github.io>) and tested our website on different operating systems and browsers, for example, MacOS + Safari, MacOS + Google Chrome, MacOS + Edge, Windows 11 + Edge, Windows 11 + Google Chrome, and Windows 11 + FireFox, we did not encounter any issues by now.

Additional things I've seen

- Line 74/85: In both ISD cutoffs are mentioned for highly disordered regions, but in one line, you write “disordered if [...] ISD < 0.5” and in the other “high structural disorder ISD > 0.5”

Response: We apologize for the typo. For disordered regions, we used ISD > 0.5.

- Line 84, 247, 248 (maybe more): PLDDT -> pLDDT

Response: We have replaced PLDDT with pLDDT throughout the revised manuscript.

- Fig. 1a: Please label the x-axis (confidence score?)

Response: We apologize for the confusion. We showed the distribution of pDockQ values in Fig. 1a. Thus, the x-axis are bins of pDockQ values. We have now labeled the x-axis using “pDockQ”.

- Fig. 1b: I know it is a network of possible protein-protein interactions, but for me as a reader, it does not show anything informative besides the network size and a lot of points over each other.

Response: We thank the reviewer for this comment. The intent of Fig. 1b is primarily to illustrate the highly connected and dense nature of the predicted protein-protein interaction (PPI) network, rather than to highlight individual connections. We agree that the overall network layout can appear visually crowded due to the large number of predicted interactions. To make this clearer, we have revised the figure legend in the revised manuscript to explicitly state this purpose. We have also included the revised figure legend as follows:

b) The PPI network of predicted high-confidence PPIs by AlphaFold2 multimer. Each node represents a protein, and edges denote predicted high-confidence interactions. Each node was colored according to the number of its partners. The dense and overlapping layout reflects the highly interconnected nature of the interactome.

- Fig. 2a/b: Three median-sized connected subnetworks are mentioned in the text (l. 202-203), but I see only two in the figure. In addition, even though the names in the networks are interesting, there is too much information in them, and in some cases, I'm not sure which label belongs to which.

Response: We have added the third cluster and revised the layout of Figure 2 to present the information more clearly while retaining key details. These subnetworks represent high-confidence interactions predicted from functional associations, one of the main focuses of this study.

- Fig 2c/d: Choosing red and blue for the coloring could confuse the reader because the matrix in e uses the same colors for another purpose.

Response: We have now revised the color scheme of the structures showed throughout the revised manuscript. Specifically, we used a colorblind-friendly color scheme, with blue (#0072B2), orange (#E69F00), and cyan (#56B4E9). With the same idea in mind, we also replaced blue-red scheme with blue-orange scheme for the PAE plot.

- Fig 2c/d: Using red and green is unfortunate for colorblind readers.

Response: As replied above, we have now revised the color scheme of the structures showed throughout the revised manuscript. Specifically, we used a colorblind-friendly color scheme, with blue (#0072B2), orange (#E69F00), and cyan (#56B4E9). With the same idea in mind, we also replaced blue-red scheme with blue-orange scheme for the PAE plot.

- Fig 2c/d: It would be nice to have pLDDt colored structures or only show one structure, because it is hard/impossible for the reader to identify high confidence predictions in this complex

Response: We apologize for the confusion. For Fig 2c, there is a homolog protein complex structure in PDB. However, there is no such structure in PDB for Fig 2d. Thus, the structure in Fig 2d is only predicted structure. We have now revised the figure legend to reflect the situation. Also, in the case of Fig 2d, we have now included pLDDT colored structures in S5 Fig along with an annotated PAE map so that readers can identify high confidence predictions in this complex.

- Fig 2c/d: Why are there residues indicated in the structures of Ran/Nup50. They are not mentioned in the text. Should they interact with each other?

Response: They are terminal residues not interacting residues. We have removed these labels to avoid further confusions as we think they are not necessary.

- Fig 2d/e: Please change the numbering of the pAE matrix to match the number of the residues mentioned in the structure/text to make it clear which parts are meant. Or color some parts differently to highlight the interface.

Response: In the revised figure, we have now numbered axis of the matrix separately based on the residue index of each protein (see revised Figure 2).

- Fig 3c: Which one is Ku80 and which Irbp?

Response: We have now edited in Figure 3c legend to show that the left column is Ku80, and right column is Irpb.

- Fig 3d: In the left panel, the Ku80 residues from 246-272 are highlighted, but in the text, 245-284 are mentioned.

Response: We thank the reviewer for pointing out this typo. We have now corrected 245-284 in the main text to 246-272 in the revised manuscript.

**Reviewer #2**

General comments

This is a carefully conducted study, which integrates structural information with data on the protein-protein interactions (PPI) network of D. melanogaster. An original aspect of this work is the investigation of the role of regions predicted to be disordered in the structures of the individual subunits and the detailed analysis of the contribution of these regions to the binding interfaces in the complex. The study deftly uses available DL-based tools (AlphaFold, AlphaFold2 multimer) to predict the structures of individual subunits and their pairwise hetero complexes; the limits of disordered regions in these structures is defined based on these tools. Data on PPIs is obtained from various well-established sources, and more particularly from STRING, which include functional associations, derived from data on co-expression and genomic context, in addition to those detected by experimental methods. Care is taken to concentrate on predicted structures with high confidence, to reduce noise in the analysis.

Results reveal important gaps between the detailed structure-based predictions and PPIs based on the evidence compiled in STRING. Notably of the ~28000 protein pairs with high confidence in STRING (≥0.9), only about a third (~29%) were predicted to form physical interaction at an acceptable confidence level based on the structural analysis. Of these, a mere 13% (~3600 pairs) were found to do so at a high confidence level. But reassuringly, the latter were found to be well supported by a reasonable physical interaction evidence in STRING. In addition, it was observed that many of the remaining 19% high confidence predicted complexes are labelled as functional associations in STRING, suggesting that a subset of such association may actually involve physically interacting protein pairs, as it has been suggested, and occasionally confirmed, for proteins in metabolic networks.

Interesting finding are also reported based on the detailed structural analysis of the contribution of IDR regions to shaping the interfaces of predicted complexes. Namely: that most of the interfaces in high confidence PPI comprises ordered as well as disordered or conditionally folded regions. This picture is complemented by a sequence conservation analysis of 15 genomes from different taxa and highlighting examples where IDR containing interfaces are conserved despite differences in disorder propensity and binding mechanism.

In conclusion, this is an interesting paper that provides new insights into the prevalent contribution of intrinsic disorder to the formation of protein complexes. It also demonstrates the value of detailed structural analyses in shaping the landscape of physically interacting proteins.

Response: We thank the reviewer very much for the detailed summary of our work and insightful comments.

In the following are some specific comments:

1- The introduction merely lists citations of some large scale – proteome wide- studies on the role of intrinsic disorder in protein-protein interactions but lacks a succinct description of such studies. Including such description should help readers better understand the contribution of the present work.

Response: Thank you to the reviewer for the helpful suggestion. We agree that a concise description of prior large-scale and conceptual studies on the role of intrinsic disorder in protein-protein interactions will improve the clarity of the Introduction and help readers better understand the contribution of the present work. We have now revised the paragraph to summarize the key insights from these works, highlighting how they revealed distinct binding mechanisms and network features of intrinsic disorder mediated interactions, before introducing our study. We have also attached the additional introduction below for your reference.

“It has been shown that disordered regions can engage in diverse binding modes, from transient fuzzy complexes to disorder-to-order transitions upon binding (15–17, 19–22). Recent experimental and computational studies further highlight conditionally folding as an important disorder-to-order binding mechanism (19, 23). Despite these studies, systematic characterization of different disorder binding modes and their contributions to protein-protein interactions at the proteome scale remains limited.”

2- Proteins frequently form homodimers, larger homo-oligomers, or quasi homo-oligomers (with paralogues). Such complexes may form different interfaces, including with other proteins. This situation was ignored here by investigating only hetero pairs formed by representative proteins! Ignoring information on paralogues is understandable since it is rarely if ever available from experimental methods or from the PPI DBs. On the other hand, the authors should check which proteins are likely to form homo-oligomers by using data from the study of Sweke et.al. (Cell 2024), which used AlphaFold to compute the atlas of homo-oligomers across the domains of life, and see what the impact might be on the results. Also, since many homo- dimers/oligomers tend to form co-translationally, it may be of interest to investigate the role of disorder in these complexes, to find out if it differs from the one in the heterodimers.

Response: We really thank the reviewer for this thoughtful idea. Yes, it would be great to see the interactions of homodimers, as they represent a non-negligible part of protein-protein interactions in cells.

For the disorder part, due to the methodology in our current analysis, we could not compute the pDockQ values from the available structures provided by Sweke et al at <https://figshare.com/s/af3c1d5969f7468f2caa>. The reason is that sometimes residues could be close in AlphaFold2 multimer predictions, but the closeness does not necessarily mean they interact. In this study, we decide if two residues interact if they are close (within 7 Å), and in the meantime, the PAE between the two residues are smaller than 15 Å. In the dataset from Sweke et al., we cannot find the PAE matrix and there are no *Drosophila* proteins, thus we cannot directly extend our current analysis to this dataset for now. However, we do think that extending our analysis to similar datasets, e.g. homo-oligomers in the Drosophila proteome, would be a valuable future direction. Thus, in discussion, we added a section as below.

“While the STRING database focuses on heterodimeric interactions, many proteins also function as homodimers or higher-order oligomers. A recent study explored the potential for homo-oligomer formation at the proteome scale using AlphaFold2 Multimer predictions (45). Extending our analysis to include such self-associations would be another valuable future direction.”

3- The described network analysis identifies several highly connected regions, which the authors report as containing many ribosomal proteins in addition to others. This is not surprising, as ribosomal proteins are often reported to ‘contaminate’ detected PPI and are therefore called ‘frequent flyers’. Due to their role in the translation machinery, they engage in many interactions, a significant fraction of which may be spurious (not evolved for function). The authors may want to delete these proteins, re-analyze the resulting network, and report the results if those differ.

Response: We thank the reviewer for bringing out this issue. We agree that some ribosomal proteins may have non-specific binding with suspicious function because they are highly positively charged. Thus, we re-analyzed the network by removing these proteins. There are 154 ribosomal proteins involved in this study, including both cytoplasmic and mitochondrial. After removing the ribosomal proteins, we observed a similar layout of the overall PPI network. We further checked the overall connectivity distribution of the modified network and found it similar to that of the original. The results suggest that, even without ribosomal proteins, the network remains highly connected, consistent with the high-confidence PPI network's connectivity. We have added this important result to S4 Fig and attached it below for your reference.

**S4 Fig. High-confidence PPI network after removing ribosomal proteins.** a) The modified network, obtained after removing 154 ribosomal proteins, including both cytoplasmic and mitochondrial, retains a similar overall layout to the original network (Fig 1b in the main text). b) The modified network also shows a comparable connectivity distribution to the original network. The connectivity distribution represents the number of proteins that have a given number of interaction partners. Note that the Y-axis is in log scale, log_2_(Number of proteins + 1).

4- AlphaFold-Disorder is only fleetingly mentioned in Ref 30 which is a review of databases of protein disorder. It probably involves regions of the polypeptide predicted with low confidence score by AlphaFold2-multimer. The exact procedure used should be succinctly outlined.

Response: We apologize for the confusion. Besides reviewing the structures in the AlphaFold2 database, the cited paper introduced a tool, AlphaFold-Disorder, to assess protein disorder, which we used to detect disordered regions in protein monomers. By doing this, we are able to detect conditionally folded regions, which have both high structural disorder and high AlphaFold confidence scores (pLDDT).

5- In line 235, it is stated that coil-to-order transition is more likely to involve induced fit. In light of the recent understanding of Allostery, which essentially rejects the induced fit as a valid mechanism, the coil-to-order transition may also involve conformational selection whereby, a very lowly populated structured conformer be selected, provided the interaction with the binding partner stabilizes it sufficiently.

Response: We appreciate the reviewer’s insightful comment regarding the mechanism underlying the coil-to-order transition. The reviewer notes that, in light of ensemble-based views of allostery, coil-to-order binding should not be framed exclusively as induced fit; rather, conformational selection may also contribute, whereby a pre-existing, very low-populated conformation is selected and then stabilized upon binding. Our rationale for stating it as “more likely to be driven by induced fit” was as follows: (1) AlphaFold generally predicts a low energy state of proteins or IDRs. The conditionally folded proteins or regions are some examples. For these regions, the proteins or IDRs may undergo dynamic conformational changes, with the predicted structures being a low energy state. Thus, for binding involved in conditionally folding, it is more likely to be conformational selection. (2) In the case where AlphaFold monomer prediction failed to capture the binding state conformation, we tend to think that the binding state conformation may not be naturally occurring, or is in a relative high energy state. In this sense, we think that the transition from coil (in monomer state) to order (in binding state) may be an extra entropy-favored mechanism, such as induced fit.

However, after considering the reviewer’s insightful comments, and the fact conformational selection and induced fit are often coupled during protein-protein interactions (Vogt AD, Di Cera E. Conformational selection or induced fit? A critical appraisal of the kinetic mechanism. Biochemistry. 2012 Jul 31;51(30):5894-902. doi: 10.1021/bi3006913), we recognized that conformational selection and induced fit are not mutually exclusive. In this sense, conformational selection towards a low populated conformation could also account for “coil-to-order” transitions and it would be more reasonable to state it as a combination of conformational selection and induced fit.

Based on the reasons above, in the revised manuscript, we have rephrased it as “CF binding was hypothesized to occur predominantly via conformational selection (23, 32), while coil-to-order binding is more likely driven by a combined mechanism of conformational selection and induced fit, where induced fit represents an entropy-favored mechanism in which a disordered region folds into a specific structure upon binding, adapting its shape to fit the interacting partner (22)”

6- I used the link provided in the text hoping to examine the interactive web interface, but unfortunately, except for the master Table on the Home page, none of the ‘view details’

links worked. The authors should make sure their interface is operational, including on a Mac, before resubmitting the revised version of their paper.

Response: We apologize the inconvenience. Now we have updated our site (https://melppi.github.io) and tested our website on different operating systems and browsers, for example, MacOS + Safari, MacOS + Google Chrome, MacOS + Edge, Windows + Edge, Windows + Google Chrome, and Windows + FireFox, we did not encounter any issues by now.

**Reviewer #3**

In this work entitled “A predicted structural interactome reveals binding interference from intrinsically disordered regions”, the authors present a large-scale prediction and systematic analysis of the Drosophila melanogaster interactome using AlphaFold2 Multimer combined with STRING functional and physical interaction datasets. Importantly, the authors provide an interactive web resource (DmelPPI).

Strength of the study is the integration of functional interactions. By not limiting the analysis to strictly physical interactions, the authors capture a broader landscape of potentially meaningful connections, many of which are supported by indirect evidence such as co-expression or text-mining. This inclusive approach, coupled with structural predictions, yields a resource that is valuable for both computational exploration and the generation of testable experimental hypotheses. The fact that many predicted complexes align well with known homologous structures adds confidence to the robustness of the pipeline. pDockQ has been used to assess AF2 prediction. Moreover, the authors place particular emphasis on the role of intrinsically disordered regions (IDRs), which they show are involved in the majority of high-confidence protein–protein interactions, often through coil-to-order transitions or conditional folding. Notably, the discrepancies obtained through the presented protocol have been carefully discussed and assessed.

The manuscript is overall well written, scientifically rigorous, and of clear relevance to the computational biology community. It offers a good contribution by not only cataloging predicted interactions but also highlighting mechanistic roles of IDRs, a feature that is often overlooked in large-scale structural interactome studies. The interactive website further enhances its value as a community resource.

I consider the work suitable for publication in PLOS Computational Biology.

Response: We truly appreciate the reviewer’s insightful comments and constructive recommendations.

Minor comments:

• As stated by the authors “While computationally predicted models often align well with experimental structures … these predictions still require careful interpretation, especially for IDRs due to their inherently dynamic and context-dependent nature.” In this context, I think it would be of useful if the authors provide guidelines/opinions on how the think the interpretation should be lead (taking into account the variability of single cases).

Response: We thank the reviewer for this thoughtful suggestion. We agree that providing guidance on the interpretation of predicted models, especially for intrinsically disordered regions (IDRs), would be valuable. We have added a short paragraph in the Discussion outlining general considerations for interpreting such predictions while acknowledging the variability among individual cases. In particular, when a predicted interaction involves an IDR, we advise caution, as such interactions may represent transient or context-dependent associations rather than stable folds. In this sense, combining confidence metrics such as pLDDT and PAE can provide useful indicators of local flexibility and uncertainty. We also noted in the original main text that developing more sophisticated, dedicated metrics could further improve the interpretation of such predictions.

• It may be useful to briefly discuss why AF2 and not AF3 has been used.

Response: We thank the reviewer for bringing this out. At the time this project was initiated, AlphaFold3, and other state-of-the art models, such as Boltz, and Chai had not yet been released. Given our limited computational resources and the fact that it took us many months to finish the current version of predictions, we focused on AlphaFold2 multimer, which has been widely validated and shown to achieve comparable accuracy to these more recent models for many protein-protein complex predictions.

• On page 6 line 38, the notation “etc.” does not give accuracy to the whole sentence. I suggest to extended the given examples.

Response: We thank the reviewer for pointing this out. We have replaced “etc” with “and underrepresentation of transient or weak interactions” to show another common limitation in detecting PPIs (Greenblatt,J.F., et al. 2024. Cell, 187, 6501–6517).
